# Dosimetric and radiobiological comparison for quality assurance of IMRT and VMAT plans

**DOI:** 10.1002/acm2.12145

**Published:** 2017-08-03

**Authors:** Nava Raj Paudel, Ganesh Narayanasamy, Eun Young Han, Jose Penagaricano, Panayiotis Mavroidis, Xin Zhang, Anil Pyakuryal, Dongwook Kim, Xiaoying Liang, Steven Morrill

**Affiliations:** ^1^ Department of Radiation Oncology University of Arkansas for Medical Sciences Little Rock AR USA; ^2^ Department of Radiation Oncology UPMC Susquehanna Williamsport PA USA; ^3^ Department of Radiation Physics The University of Texas MD Anderson Cancer Center Houston Texas USA; ^4^ Department of Radiation Oncology University of North Carolina Chapel Hill NC USA; ^5^ University of District of Columbia Washington DC USA; ^6^ Department of Radiation Oncology Kyung Hee University Hospital at Gangdong Seoul Korea; ^7^ Department of Radiation Oncology University of Florida Gainesville FL USA

**Keywords:** 3DVH, ArcCheck, dosimetric quality assurance, NTCP, radiobiological QA, TCP

## Abstract

**Introduction:**

The gamma analysis used for quality assurance of a complex radiotherapy plan examines the dosimetric equivalence between planned and measured dose distributions within some tolerance. This study explores whether the dosimetric difference is correlated with any radiobiological difference between delivered and planned dose.

**Methods:**

VMAT or IMRT plans optimized for 14 cancer patients were calculated and delivered to a QA device. Measured dose was compared against planned dose using 2‐D gamma analysis. Dose volume histograms (for various patient structures) obtained by interpolating measured data were compared against the planned ones using a 3‐D gamma analysis. Dose volume histograms were used in the Poisson model to calculate tumor control probability for the treatment targets and in the Sigmoid dose–response model to calculate normal tissue complication probability for the organs at risk.

**Results:**

Differences in measured and planned dosimetric data for the patient plans passing at ≥94.9% rate at 3%/3 mm criteria are not statistically significant. Average ± standard deviation tumor control probabilities based on measured and planned data are 65.8±4.0% and 67.8±4.1% for head and neck, and 71.9±2.7% and 73.3±3.1% for lung plans, respectively. The differences in tumor control probabilities obtained from measured and planned dose are statistically insignificant. However, the differences in normal tissue complication probabilities for larynx, lungs‐GTV, heart, and cord are statistically significant for the patient plans meeting ≥94.9% passing criterion at 3%/3 mm.

**Conclusion:**

A ≥90% gamma passing criterion at 3%/3 mm cannot assure the radiobiological equivalence between planned and delivered dose. These results agree with the published literature demonstrating the inadequacy of the criterion for dosimetric QA and suggest for a tighter tolerance.

## 1 INTRODUCTION

Treatment plans used in radiation therapy are generally evaluated on the basis of dose distribution and dose volume parameters. Accuracy of treatment delivery of complex treatment plans is assured through a quality assurance (QA) process using ion chambers, films and more commonly with diode array,^1^, ^2^ and ion chamber array measurements.^3^ Measured data are generally compared against planned data using two‐dimensional (2‐D) gamma analysis. In reality, treatment target and organs at risk (OARs) present 3‐D geometry, and a 2‐D gamma analysis‐based dosimetric comparison may not provide information about the criticality of a disagreement. Several studies^4^, ^5^, ^6^, ^7^ have shown that 2‐D gamma analysis fails to detect errors in some cases. Even though detailed analysis is still under investigation,^8^ dose volume histogram (DVH)‐based dosimetric evaluation can provide structure‐by‐structure information and a 3‐D gamma analysis can be a better option for the QA purpose. Validity of DVH‐based evaluation of delivered intensity‐modulated radiation therapy (IMRT) plans against the corresponding plans optimized with treatment planning system (TPS) have been demonstrated using film, ion chamber,^9^ and BANG3 gel dosimetry.^10^ A number of studies show that a DVH‐based 3‐D gamma analysis provides more reliable comparison than a point‐by‐point per‐beam 2‐D gamma analysis of IMRT plans.^8^, ^11^, ^12^

Most of the DVH‐based studies were based on measurement and/or interpolation of 2‐D device measured data into 3‐D dose distribution.^7^, ^10^, ^11^ The DVH comparison studies have shown differences between planned and measured DVHs as well as differences in mean doses.^10^, ^13^ As per the report of Task Group (TG) 65 of American Association of Physicists in Medicine (AAPM), tumor control probabilities (TCPs) are 2–4 more sensitive with respect to the change in uniform dose and normal tissue complication probabilities (NTCPs) are and 4–6 times more sensitive to the change in uniform dose.^14^ However, none of the above mentioned studies have evaluated whether radiobiological differences existed in any of the cases. A growing recognition of the limitation of dose volume parameters in correlating with biological response has prompted for the use of radiobiological models for treatment planning^15^ but QA of all plans is still performed on the basis of dosimetric comparison alone. Very recently, an attempt for a radiobiological comparison between delivered and planned IMRT treatment plans was made using a 2‐D QA device (MapCheck, Sun Nuclear) measured dose.^16^

Here, we use a cylindrical QA device, ArcCheck, for the measurement and compare the measured data against TPS‐calculated (planned) data using 2‐D gamma analysis in SNC Patient^TM^ software. Application of ArcCheck for patient‐specific dosimetric QA^12^ and DVH‐based plan verification using Sun Nuclear's 3DVH^®^ software has been experimentally verified elsewhere.^17^ In this study, DVHs for various structures were created from the ArcCheck measured data using 3DVH software and compared against the TPS‐planned DVHs using 3‐D gamma analysis. Radiobiological comparison of ArcCheck measured plans was performed against the corresponding TPS‐optimized volumetric‐modulated arc therapy (VMAT) or static gantry IMRT plans.

## 2 MATERIALS AND METHODS

### 2.A Patient selection, treatment planning, and measurement

Fourteen patients treated with IMRT or VMAT were retrospectively selected for this study. Among them, seven were head and neck (H&N) patients and seven were lung patients. Three of H&N patients were planned for VMAT and four were planned for IMRT. Similarly, five of the lung patients were planned for VMAT and two were planned for IMRT. VMAT plans used two full or partial arcs while IMRT plans used 7–9 static fields to get optimal target coverage. Sliding window method was used in all plans. Computed tomography (CT) simulation was performed in a Philips Big Bore CT scanner (Philips Medical Systems, Amsterdam, Netherlands). Vac‐Locs and type‐S masks (CIVCO Radiotherapy, Coralville, Iowa) were used for immobilizing lung and H&N patients, respectively. Varian couch is modeled in our TPS and was inserted in each treatment plan. H&N mask was included in the body contour (used for dose calculation) while Vac‐Loc was not included in the body contour. Even though the beam attenuation due to the immobilization devices is minimal, the field arrangement was optimized to minimize the fraction of the beams passing through them. The lung plans were optimized with Acuros XB algorithm and all other plans were optimized with AAA algorithm in Eclipse TPS (Version 11.0.47, Varian Medical Systems, Palo Alto, CA, USA) using 6 MV beam of a Varian TrueBeam STX linear accelerator. Dose of 66 Gy was prescribed to gross tumor volume (GTV) of all H&N patients while 60 Gy and 54 Gy were prescribed to lymph nodes for a few patients. Five of seven lung patients were prescribed to 66 Gy and other two were prescribed to 61.2 and 72 Gy, respectively. The dose grid resolution used was 1 mm for lung and 2 mm for H&N plans. All the plans were normalized to cover 95% of target volume by 100% of the prescribed dose while minimizing dose to the surrounding OARs. The planning objectives in terms of dose constraints to OARs were the following: maximum dose to the spinal cord 50 Gy, maximum dose to the brainstem 60 Gy, mean dose to parotids <26 Gy (or median dose <30 Gy), mean dose to larynx <40 Gy, mean dose to heart <20 Gy, and mean lung dose <20 Gy and V_20 Gy_ <25% to the lungs. In few patients, some of the OAR dose constraints were not met due to the close proximity of infiltration of the tumor to those OARs. The optimized plan doses were calculated on ArcCheck images in Eclipse TPS and delivered to the ArcCheck phantom (Sun Nuclear, Melbourne, FL, USA) with the insert in place. Cumulative dose was recorded using the SNC Patient software version 6.2.1 (Sun Nuclear, Melbourne, FL, USA).

### 2.B Dosimetric comparison

Dosimetric comparison between planned and measured data comprised of mean and maximum dose to treatment target and few OARs. For H&N plans, mean dose to GTV, esophagus, larynx, parotids, and maximum dose to brainstem and cord were compared while for lung plans, mean dose to the GTV, heart, esophagus, normal lung, and maximum dose to the cord were compared.

The measured data were compared against the planned data using a minimum of 90% pass rate with 2‐D gamma criteria of 3 mm distance to agreement (DTA), 3% dose difference (DD) global^1^ using the SNC Patient. A 10% dose threshold and global normalization was used.

The 3DVH software requires two set of data for comparison. TPS‐optimized patient plan, dose distribution, contoured structures and planning CT images of each patient, corresponding TPS‐calculated (planned) dose on ArcCheck CT images, and measured data were imported to 3DVH software version 3.2. The ArcCheck measured data were then converted into 3‐D dosimetric data with planned dose perturbation (PDP) algorithm^8^, ^9^ and compared against original patient data obtained from TPS using the 3‐D gamma analysis (3 mm DTA, 3% DD global). The 3‐D gamma analysis results were compared against the corresponding 2‐D gamma analysis results. DVH data for target and various structures obtained from 3DVH and TPS (1 cGy bin size) were imported to MATLAB (The MathWorks, Inc., Natick, MA, USA) for radiobiological comparison.

### 2.C Radiobiological comparison

DVHs based on measurement and TPS were used in biological models to calculate TCPs and NTCPs using MATLAB. Conventional fractionation scheme (1.8–2 Gy per fraction) was used for the calculations.

### 2.D TCPs with Poisson model

Clinical target volume (CTV) is the volume of tumor intended to treat. In our study, GTV included gross tumor and subclinical microscopic disease, and CTV was labeled as GTV. Hence, it is appropriate to calculate TCP for GTV even though dose is prescribed to planning target volume (PTV) to incorporate set up error. In this study, TCPs for H&N and lung GTVs were calculated using the Poisson model,^18^, ^19^, ^20^ which is expressed in eq. (1).

(1) $$TCP={\left[\frac{1}{2}\right]}^{\sum_{i}v_{i}\exp\left\{2\gamma_{50}\left(1-\frac{D_{i}}{D_{50}}\right)/ln2\right\}}$$

Here, D_50_ is dose yielding 50% probability for tumor control and *γ*_50_ is slope of the dose–response curve at the level of 50% TCP. Similarly, D_i_ and v_i_ are the dose and volume elements of DVHs, respectively.

For the calculation purpose, 63.43 Gy and 51.24 Gy were used as D_50_ for H&N GTV and lung GTV, respectively. Similarly, the values of 2.66 and 0.83 were used, respectively, for *γ*_50_. These values were derived from clinical data (DVHs and treatment outcomes) using a cohort of 90 patients. A regression analysis was used to fit the TCP model into the clinical data.^21^

### 2.E NTCP with sigmoid dose–response model

The values of NTCP for various OARs were calculated using DVHs obtained from planned and measured data in the sigmoid dose–response (SDR) model,^21^, ^22^, ^23^ expressed in eq. (2).

(2) $$NTCP=\Phi \left(\frac{EUD-D_{50}}{mD_{50}}\right)$$

Here, Φ is the probit function defined by:

(3) $$\begin{array}{cc} \Phi \left(x\right) & =\frac{1}{\sqrt{2\pi }}\int_{-\infty }^{x}\exp\left(-\frac{t^{2}}{2}\right)dt\\ & =\frac{1}{2}\left[1+\mathrm{erf}\left(\frac{x}{\sqrt{2}}\right)\right] \end{array}$$

Here, x = (EUD – D_50_)/mD_50_, where D_50_ is the dose yielding 50% NTCP, obtained from dose–response curve, and EUD is equivalent uniform dose, defined as the dose which distributed uniformly over a structure would produce the same effect as the dose specified by the DVH. The parameter m represents the slope of the dose–response curve. EUD is also defined as a generalized equivalent uniform dose (gEUD) calculated using the series of dose volume pairs (D_i_. v_i_), obtained from the DVH of a structure using the formula expressed in eq. (4).

(4) $$gEUD={\left[\sum_{i}v_{i}D_{i}^{1/n}\right]}^{n}$$

Here, n is a parameter that determines the dose volume dependence of a given OAR.

Brainstem, spinal cord, esophagus, larynx, and left parotid and right parotid NTCPs were evaluated for H&N plans while bilateral lungs, heart, spinal cord, and esophagus NTCPs were evaluated for lung patients. D_50_, n, and m values from Burman et al.^22^, ^24^ used for NTCP calculations are tabulated in Table 1.

**Table 1:** D_50_, m, and *n* values used to calculate NTCPs

Structure	Larynx	Esophagus	Parotids	Brainstem	Cord	Bilat. lungs	Heart
D_50_ (Gy)	70.0	68.0	46.0	65.0	66.5	24.5	48.0
m	0.17	0.11	0.18	0.14	0.17	0.18	0.10
n	0.08	0.06	0.70	0.16	0.05	0.87	0.35

### 2.F Statistical analysis

The Shapiro–Wilk test was used to test the normality of the data. A two‐tailed Student *t*‐test (0.05 significance level) was performed for the data following normal distribution to test the significance in difference between (a) planned and measured dose to the studied structures, (b) 2‐D and 3‐D gamma analysis results, (c) TCPs based on planned and measured data, and (d) NTCPs based on planned and measured data. Wilcoxon signed rank test was performed on the data not following a normal distribution.

### 2.G Effect of tighter tolerance on dosimetric analysis

Tighter tolerances of 2.5%/2.5 mm and 2%/2 mm global normalization were used for 2‐D gamma analysis to check whether a radiobiological equivalence between planned and delivered dose distributions exists for the patient plans passing at ≥90%.

## 3 RESULTS

### 3.A Dosimetric comparison

Mean and maximum doses to GTV and various OARs for H&N and lung patients are tabulated in Tables 2 and 3, respectively. Also the p‐values of the statistical test between planned and measured dose for each structure are included in the tables.

**Table 2:** Mean or maximum dose (Gy) to various structures obtained from treatment plan and measurement, and *P*‐values of statistical test for H&N patient plans

Patient No.		Mean dose (Gy)			Lt Parotid	Rt Parotid	Maximum dose (Gy)	
		GTV	Larynx	Esophagus			Brainstem	Cord
1	Planned	67.8	47.6	30.0	28.9	27.5	23.4	45.8
	Measured	69.4	48.4	30.3	29.2	27.5	22.4	46.0
2	Planned	65.4	32.3	36.7	25.0	27.8	29.3	30.9
	Measured	66.0	32.7	36.6	25.5	28.3	29.7	31.3
3	Planned	67.4	38.8	31.9	25.9	23.7	39.8	46.6
	Measured	69.1	39.7	32.6	26.3	24.1	41.1	47.5
4	Planned	67.5	37.4	1.0	62.0	21.8	24.8	12.6
	Measured	67.2	37.6	1.0	61.2	21.6	24.9	12.2
5	Planned	68.7	7.5	0.4	1.4	1.2	0.6	20.0
	Measured	68.8	8.1	0.4	1.5	1.3	0.7	10.8
6	Planned	68.0	64.6	10.9	28.5	29.1	23.2	50.4
	Measured	68.8	66.5	11.0	28.4	29.1	23.4	46.2
7	Planned	69.1	40.2	34.1	25.3	32.5	50.7	47.8
	Measured	69.1	40.7	34.1	24.9	32.2	51.2	48.5
Average ± SD	Planned	67.7 ± 1.2	38.3 ± 17.2	20.7 ± 16.0	28.1 ± 17.7	23.4 ± 10.4	27.4 ± 15.6	36.3 ± 15.2
	Measured	68.3 ± 1.3	39.1 ± 17.5	20.9 ± 16.1	28.1 ± 17.4	23.4 ± 10.4	27.6 ± 15.9	34.6 ± 16.8
*P*‐value		>0.05	>0.05	>0.05	>0.05	>0.05	>0.05	>0.05

**Table 3:** Mean or maximum dose (Gy) to various structures obtained from treatment plan and measurement, and p‐values of statistical test for lung patient plans

Patient No.	Dose (Gy)	GTV_mean_	Heart_mean_	Cord_max_	Esophagus_mean_	Lung_mean_
1	Planned	67.6	13.9	40.9	21	18.9
	Measured	69.6	14.6	41.9	21.7	19.6
2	Planned	63.4	3.4	39.1	19.7	16.2
	Measured	63.9	3.5	39.5	19.9	16.4
3	Planned	68.1	0.3	28.1	3.3	3.5
	Measured	67.3	0.3	29	3.4	3.6
4	Planned	67.8	8.7	43.4	29.8	16.3
	Measured	69.3	8.8	44.4	29.8	16.9
5	Planned	70.2	1.7	43.7	6.7	13.3
	Measured	73	1.8	47	6.7	13.8
6	Planned	64.8	4.7	37	15.7	13.2
	Measured	66.3	4.8	37.4	15.8	13.6
7	Planned	69.2	1.2	36.8	16.2	8.5
	Measured	70.6	1.2	37.3	16.6	8.8
Average± SD	Planned	67.3 ± 2.4	4.8 ± 4.9	38.4 ± 5.3	16.1 ± 8.9	12.8 ± 5.3
	Measured	68.6 ± 3.0	5.0 ± 5.1	39.5 ± 5.9	16.3 ± 9.0	13.2 ± 5.4
*P*‐value		>0.05	>0.05	>0.05	>0.05	>0.05

As signified by the *P*‐values of statistical test tabulated in Tables 2 and 3, the differences between planned and measured dose are not statistically significant at 0.05 significant level for any of the structures studied.

**Figure 1:**
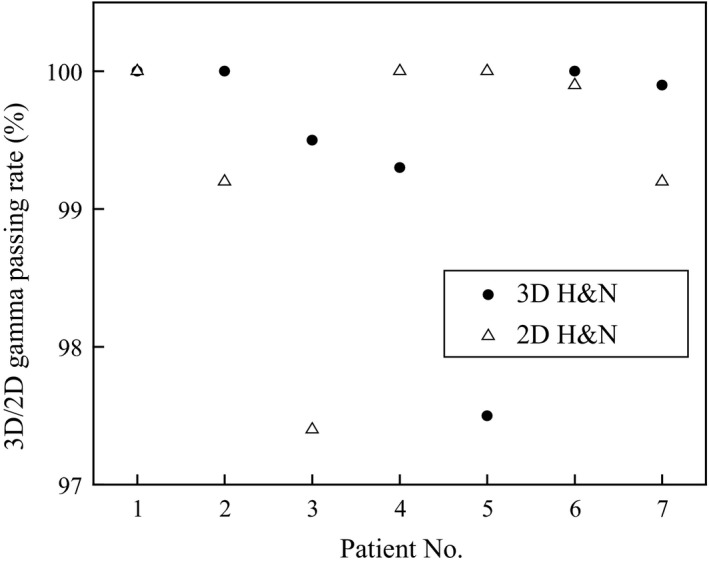
Two‐D and 3‐D gamma pass rates for H&N patient plans (patients 4, 5, & 7 are VMAT and all others are IMRT).

**Figure 2:**
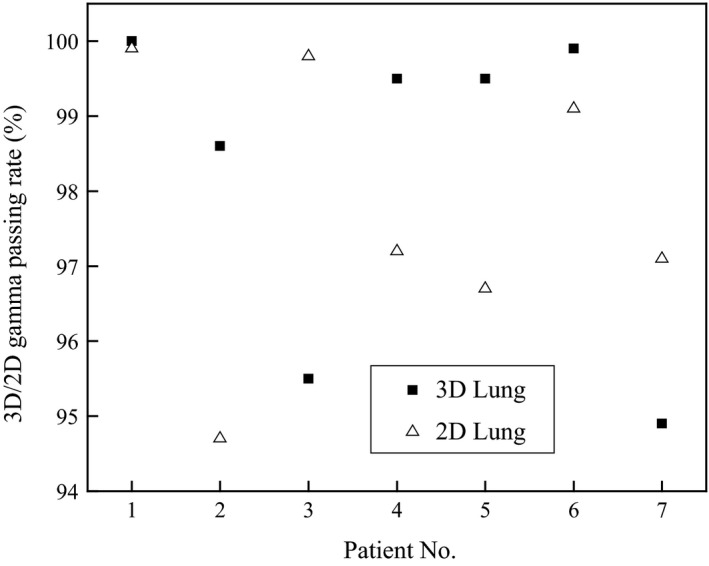
Two‐D and 3‐D gamma pass rates for lung patient plans (patients 1 & 6 are IMRT and all others are VMAT).

Similarly, the results of 2‐D and 3‐D gamma analysis are compared in Figures 1 and 2.

The differences in 2‐D and 3‐D gamma passing rates ranged between 0% and 2.7% for H&N patient plans with a mean difference of 0.1% while they ranged between 0.1% and 4.5% for lung patient plans with a mean difference of 0.5%. However, a statistical test performed on both H&N and lung patient plans showed no significant differences between 2‐D and 3‐D gamma analysis results at 95% confidence level. *P*‐values of the test were >0.05 for both H&N and lung patient plans.

**Figure 3:**
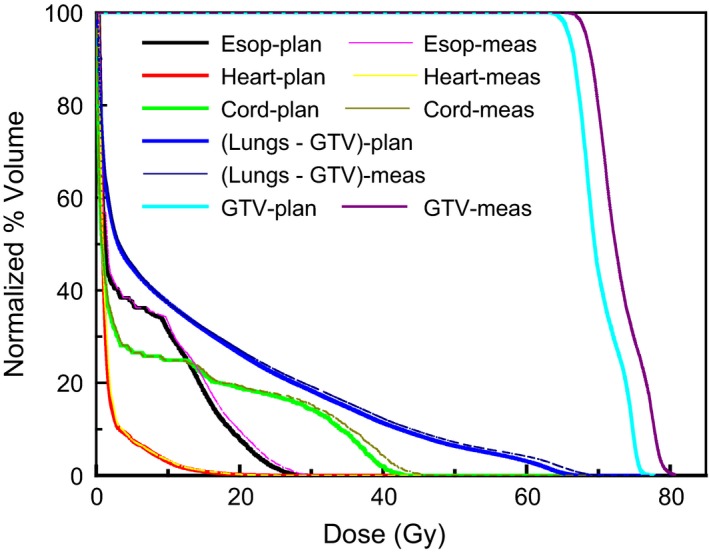
DVHs obtained from treatment plan and ArcCheck measured data for a lung patient plan.

A DVH comparison based on planned and measured data for a patient (patient 2, Figure 2) plan is presented in Figure 3.

### 3.B Radiobiological comparison

#### 3.B.1 TCP comparison

Mean and standard deviation (SD) of TCPs for patient plans, and *P*‐values of statistical test between the dataset based on plan and measurement are tabulated in Tables 4 and 5.

**Table 4:** TCPs and statistical values from planned and measured data for H&N patients

Patient No.		1	2	3	4	5	6	7	Mean	SD	*P*‐value
TCP (%)	Planned	57.9	64.7	65.0	66.5	67.0	69.2	70.1	65.8	4.0	>0.05
	Measured	60.2	63.8	70.3	71.2	69.5	69.6	70.0	67.8	4.1	

**Table 5:** TCPs and statistical values from planned and measured data for lung patients

Patient No.		1	2	3	4	5	6	7	Mean	SD	*P*‐value
TCP (%)	Planned	67.5	69.1	72.4	72.7	72.9	74.1	74.9	71.9	2.7	>0.05
	Measured	68.2	70.8	74.5	74.2	72.1	75.5	77.6	73.3	3.1	

As evident from Tables 4 and 5, the differences between two set of TCPs are statistically insignificant for H&N as well lung patients plans.

#### 3.B.2 NTCP comparison

The EUDs to majority of the OARs calculated based on the planned DVHs and measured data were close to each other and the differences were insignificant for majority of H&N as well as lung patient OARs. However, the differences were significant in few cases. There was a significant difference in NTCPs for larynx in H&N patients and for lungs‐GTV, cord, and heart in lung patients. NTCPs from planned and measured data as well as *P*‐values of the statistical test for the studied OARs are tabulated in Tables 6 and 7, respectively. The structures with significant difference in NTCPs are marked bold.

**Table 6:** NTCPs from planned and measured data for H&N patient OARs and *P*‐values of statistical test

Structure	NTCP (%)	Patient No.							Average ± SD	*P*‐value
		1	2	3	4	5	6	7		
Brainstem	Planned	<0.5	<0.5	<0.5	<0.5	<0.5	<0.5	<0.5	<0.5 ± <0.5	>0.05
	Measured	<0.5	<0.5	<0.5	<0.5	<0.5	<0.5	<0.5	<0.5 ± <0.5	
Cord	Planned	1.6	1.1	0.7	<0.5	<0.5	<0.5	<0.5	0.5 ± 0.6	>0.05
	Measured	1.7	1.3	0.8	1.1	<0.5	<0.5	<0.5	0.7 ± 0.7	
Esophag.	Planned	0.7	1.5	<0.5	<0.5	<0.5	<0.5	< 0.5	0.5 ± 0.6	>0.05
	Measured	0.8	1.8	<0.5	<0.5	<0.5	<0.5	< 0.5	0.5 ± 0.6	
**Larynx**	Planned	2.0	3.8	37.1	0.9	<0.5	<0.5	14.7	8.4 ± 13.6	**<0.05**
	Measured	2.4	4.4	44.4	1.2	<0.5	<0.5	15.8	9.8 ± 16.2	
Lt Parotid	Planned	1.1	<0.5	<0.5	<0.5	1.2	<0.5	56.7	8.5 ± 21.3	>0.05
	Measured	0.9	<0.5	<0.5	<0.5	1.4	<0.5	52.0	7.8 ± 19.5	
Rt Parotid	Planned	9.2	<0.5	<0.5	<0.5	2.7	<0.5	< 0.5	1.7 ± 3.4	>0.05
	Measured	8.6	<0.5	<0.5	<0.5	3.1	<0.5	< 0.5	2.5 ± 4.0	

**Table 7:** NTCPs from planned and measured data for lung patient OARs and *P*‐values of statistical test

Structure	NTCP (%)	Patient No.							Average ± SD	*P*‐value
		1	2	3	4	5	6	7		
**Lungs‐GTV**	Planned	15.6	1.3	1.4	<0.5	4.9	6.7	<0.5	4.3 ± 5.6	**<0.05**
	Measured	20.0	1.5	2.0	<0.5	6.4	7.4	<0.5	5.3 ± 7.1	
**Heart**	Planned	<0.5	<0.5	<0.5	<0.5	<0.5	<0.5	<0.5	<0.5 ± <0.5	**<0.05**
	Measured	<0.5	<0.5	<0.5	<0.5	<0.5	<0.5	<0.5	<0.5 ± <0.5	
**Cord**	Planned	<0.5	<0.5	<0.5	<0.5	<0.5	<0.5	<0.5	<0.5 ± <0.5	**<0.05**
	Measured	<0.5	<0.5	0.5	<0.5	0.5	<0.5	<0.5	2.3 ± 5.5	
Esophag	Planned	2.5	<0.5	<0.5	<0.5	28.4	1.3	<0.5	4.6 ± 10.5	>0.05
	Measured	4.9	<0.5	<0.5	<0.5	34.5	1.6	<0.5	5.9 ± 12.8	

NTCPs for the other OARs in most of the studied patients were negligible, ranging between 10^−7^% and less than a percent. Only the values greater than or equal to 0.5% have been tabulated to one significant figure in Tables 6 and 7, and all values smaller than 0.5% have been indexed as <0.5%.

As evident from Tables 6 and 7, differences in NTCPs based on measurement and calculation are statistically significant for larynx, lungs‐GTV, heart, and cord. Even though the values are a few percent, the statistically significant difference in NTCPs may indicate a possibility of clinically significant difference between delivered and intended outcome.

### 3.C Effect of tighter tolerance on dosimetric gamma analysis

A 2‐D gamma analysis performed at 2.5%/2.5 mm reduced the pass rate slightly for most of the patient plans but not below 92.7% for any of the plans. The pass rate ranged between 92.7% and 100.0% with median pass rate of 99.4% for H&N plans and between 91.0% and 99.5% with median pass rate of 96.6% for lung plans. However, for a 2‐D gamma analysis based on 2%/2 mm criterion, pass rate for H&N patients ranged between 85.3% and 99.6% with the median pass rate of 98.3%. For lung patient plans, it ranged between 83.3% and 97.1% with the median pass rate of 90.2%. Only five H&N patient plans and three lung patient plans met the passing criterion of ≥90% at 2%/2 mm. The statistical test on the plans passing by ≥90% at 2%/2 mm criterion did not show any radiobiological difference for any of the structures studied.

## 4 DISCUSSION

Our study showed small dosimetric differences between 2‐D and 3‐D gamma analysis results, which are in line with the results obtained by Infusino et al.^17^ using ArcCheck for the measurement. However, our radiobiological comparisons do not agree with the results from Sumida et al.^16^ where the TCPs based on measured data were found to be significantly smaller and NTCPs to be significantly higher than the ones based on planned data. Possible differences could be because of differences in device type, geometry, differences in measurement and analysis techniques, as well as the different radiobiological models used to calculate TCPs and NTCPs. While Sumida et al. had used per‐beam analysis using MapCheck measured data, we have used cumulative dose analysis using ArcCheck measured data. Due to nonzero detecting threshold of detectors used in the measurement, per‐beam analysis suppresses low dose for every beam data while with the cumulative analysis, low doses from multiple beams can add up to be detectable enough by the detectors. Also ArcCheck measures both entrance and exit doses essentially doubling the detector density while Mapcheck measures only the entrance dose. Sumida et al. had used voxel‐based Niemierko's model to calculate TCPs and NTCPs but we have used Poisson model for TCP calculation and SDR model for NTCP calculation. The other difference is that the study is based on structure‐by‐structure gamma analysis but we have performed overall gamma analysis. However, the D_50_ and *γ*_50_ values used to calculate TCPs for H&N patients in our study match with the values used in the study.

The accuracy of ArcCheck measurement and PDP algorithm is out of scope of this study. Study by Sun Nuclear Corporation shows an excellent accuracy of PDP^25^ and the algorithm has been applied in a number of independent studies^8^, ^9^, ^10^, ^11^, ^12^ before and validated against ion chamber, diode array, and gel dosimetry.

AAPM TG 119 recommends a passing criteria of 90% with 3%/3 mm DTA for per‐beam analysis and 88%–90% for composite dose analyzed with radiographic films. The minimum pass rate in our study was 94.9% at 3%/3 mm DTA for composite analysis. In spite of 94.9% minimum pass rate, the differences in NTCPs based on planned and measured data for few of the structures studied were statistically significant. However, for the patients with passing rate of ≥90% at 2%/2 mm, no statistically significant radiobiological difference was observed for any of the structures. As the number of patient plans passing by ≥90% at 2%/2 mm is small, it may not be a wise idea to generalize these results and hence a study on a large number of patients is required to determine whether 90% gamma passing rate at 2%/2 mm is sufficient for radiobiological assurance. However, our results demonstrating a statistically significant radiobiological difference for the patient plans meeting ≥94.9% pass rate at 3%/3 mm cohere with the inadequacy of 3%/3 mm gamma analysis criterion as discussed by Cadman,^26^ demonstrated by Nelms et al.^12^ and outlined by AAPM Medical Physics Practice Guideline 5.a.^27^ Our future study will focus on finding the tolerance criteria for the radiobiological quality assurance.

Another point worth mentioning is that the dosimetric or biological pass rate was not favored by any of the IMRT or VMAT modality. Although this finding is clear in our dataset, generalizing this argument is not in the scope of this study. The investigation of this topic would require a study of a different design, where other elements of the plans such as the level of beam modulation, etc., would be thoroughly studied.

## 5 CONCLUSIONS

Differences between 2‐D and 3‐D gamma analysis results for H&N and lung patients are small and statistically insignificant. The differences between TCPs obtained from the planned and measured data are also small and insignificant. However, the differences in NTCPs based on planned and measured data for a few of the structures studied are statistically significant even though the dosimetric agreements are ≥94.9% at 3%/3 mm DTA. Our study based on 14 patients suggests that ≤94.9% pass rate at 3%/3 mm DTA used for 2‐D or 3‐D gamma analysis cannot assure the radiobiological equivalence between a delivered and the corresponding planned dose. Hence, radiobiological analysis in addition to dosimetric comparison may have to be considered for the QA of complex radiotherapy plans.

## CONFLICT OF INTEREST

The authors declare no conflict of interest.
